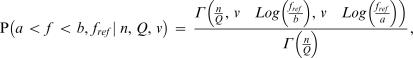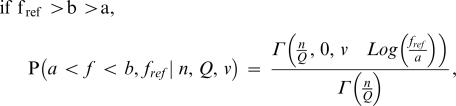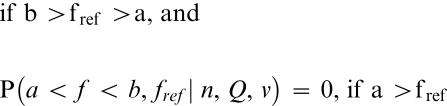# Quantifying Organismal Complexity using a Population Genetic Approach

**DOI:** 10.1371/journal.pone.0000217

**Published:** 2007-02-14

**Authors:** Olivier Tenaillon, Olin K. Silander, Jean-Philippe Uzan, Lin Chao

**Affiliations:** 1 Institut National de la Santé et de la Recherche Médicale (INSERM) U722, Faculté de Médecine Xavier Bichat, Université Denis Diderot-Paris VII, Paris, France; 2 Division of Biology, University of California San Diego, La Jolla, California, United States of America; 3 Eidgenössische Technische Hochschule (ETH) Zurich, Ecology and Evolution, ETH-Zentrum NW, Zurich, Switzerland; 4 Institut d'Astrophysique de Paris, Université Pierre et Marie Curie-Paris VI, Centre National de la Recherche Scientifique (CNRS) UMR 7095, Paris, France; Oxford University, United Kingdom

## Abstract

**Background:**

Various definitions of biological complexity have been proposed: the number of genes, cell types, or metabolic processes within an organism. As knowledge of biological systems has increased, it has become apparent that these metrics are often incongruent.

**Methodology:**

Here we propose an alternative complexity metric based on the number of genetically uncorrelated phenotypic traits contributing to an organism's fitness. This metric, phenotypic complexity, is more objective than previous suggestions, as complexity is measured from a fundamental biological perspective, that of natural selection. We utilize a model linking the equilibrium fitness (drift load) of a population to phenotypic complexity. We then use results from viral evolution experiments to compare the phenotypic complexities of two viruses, the bacteriophage X174 and vesicular stomatitis virus, and to illustrate the consistency of our approach and its applicability.

**Conclusions/Significance:**

Because Darwinian evolution through natural selection is the fundamental element unifying all biological organisms, we propose that our metric of complexity is potentially a more relevant metric than others, based on the count of artificially defined set of objects.

## Introduction

A persistent question in biology is how organismal complexity changes through the course of evolution [1]–[5]. Although significant progress has been made in the understanding and quantifying organismal characteristics at many level of organization (DNA, proteins, metabolic networks, cellular organization, organ functions, individual behavior) much confusion remains about how to accurately quantify organismal complexity. Several intuitive proposals have been made that take into account simple metrics such as the number of genes or cell types. However, these simple measures quickly lead to conflicting conclusions [2], [3]. Here we propose a fundamentally different approach to measuring organismal complexity; as opposed to relying on bottom-up measures such as the number of genes an organism has, we utilize an objective biological approach: natural selection. Instead of asking how complex an organism is from our own perspective, we ask: how complex is an organism from the perspective of natural selection? Essentially, this is a top-down metric of organismal complexity that we term phenotypic complexity.

Phenotypic complexity quantifies the number of genetically uncorrelated phenotypic traits contributing to an organism's fitness. A phenotypic trait contributes to an organism's fitness only to the extent that natural selection acts upon that trait. Thus an organismal phenotype that is no longer under selection (for example during an evolutionary transition from a generalist to specialist lifestyle), although expressed by the organism, contributes nothing to organismal complexity. Secondly, if two phenotypes contribute to complexity, they must be genetically separable: some mutations must exist that affect one phenotype but not the other. If no such mutations exist, then although we may perceive two phenotypes under selection, these phenotypes contribute only a single trait toward determining phenotypic complexity. As an example consider the affinity of an enzyme for a substrate, and the rate at which that substrate is converted to product. If there are no mutations that affect one of these traits but not the other, then these two phenotypes are considered one, until the organism gains the genetic complexity to generate variation in one phenotype without affecting the second, for example by evolving functionally separate domains in the enzyme. Phenotypic complexity is thus a combined description of how natural selection perceives organismal phenotypes and how phenotypic variation is generated by the organism. This concept was first articulated by Orr, and followed later by others [6]–[8]. Notably, it is similar to physical complexity, which is a measure of the amount of information that an organism encodes about its environment [4], [5].

An important aspect of measuring complexity in this manner is that both the organism and the environment affect the metric. An organism with many phenotypes, but living in simple environment could thus be just as complex as a simpler organism in the same environment. For example, if one organism is capable of metabolizing both lactose and glucose, while second can metabolize only glucose, the first organism will only be designated as more complex when there is a possibility that lactose will be present in the environment.

Recent population genetic theory [9], [10] has suggested that phenotypic complexity is proportional to the drift load, a quantity that describes how equilibrium mean population fitness declines with population size. This can be intuitively understood in the following manner. An organism's fitness is a result of how successfully it interacts with the environment. As the number of interactions (traits) increases, it becomes difficult to simultaneously maintain each one. Very large populations maintain nearly optimal mean population fitness regardless of phenotypic complexity. Small populations can maintain high average fitness only when there are a small number of traits (low complexity) each contributing a relatively large amount to fitness; if there are a large number of traits (high complexity), each contributing only a small amount to fitness, then small populations will be incapable of maintaining all of them. Thus for complex organisms, there will be a large difference in mean fitness between small and large populations; for simple organisms, this difference in average fitness will be small.

**Figure 1:**
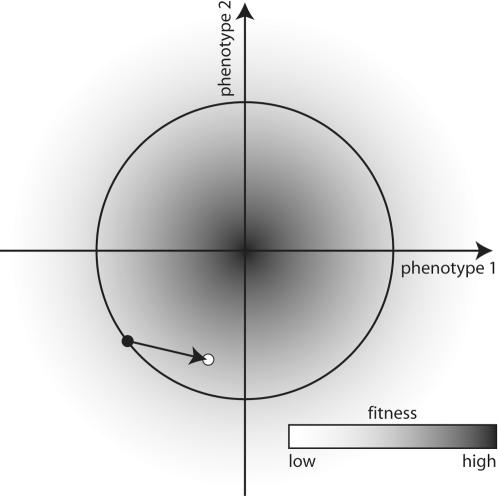
Fisher's geometric model in two-dimensional phenotypic space. Fitness varies along two phenotypic axes, with the maximum fitness located, for convenience, at the origin of these axes. Any individual in a population (black point) can thus be described by its phenotypic values, which determine the fitness of that organism. At any specific fitness, there are a number of other phenotypic combinations that have equivalent fitness; the values of these phenotypic combinations establish the fitness isoclines (black circle). From the optimum, fitness declines monotonically according to the structure of the landscape (see text). Each mutation (arrow) is drawn from a distribution centered on the phenotypic position of each individual, resulting in offspring with new phenotypic combinations and fitness values (white point).

The phenotypic model used to link drift load to phenotypic complexity was first formalized by R. A. Fisher [11]. He envisioned a multidimensional phenotypic space in which the origin of each axis corresponded to the most-fit or optimum phenotypic value. As phenotypic values depart from the origin, fitness decreases. The manner in which fitness declines is described by a monotonically decreasing function, which may take a variety of shapes, e.g., linear, concave-up, or –down. Individuals of equal fitness but different phenotypic values trace out fitness isoclines in the space. If an organism has only two phenotypes, the phenotypic space is two-dimensional and the fitness isoclines are a series of circles centered on the origin of the axes (Fig. 1). If an organism has n number of phenotypes, phenotypic space is n-dimensional, and each fitness isocline is an n-dimensional hyper-sphere. Because of the assumed geometry of the isoclines, this model is referred to as Fisher's Geometric Model (FGM) of phenotypic or adaptive evolution.

A population of individuals can be represented as a collection of points in FGM and the phenotypic values of each point allow ascertainment of the fitness of each individual. Individual fitness then determines the probability of each individual surviving and reproducing the next generation. Evolution is thus described in FGM by following the collection of points over many generations. To generate novel genetic variation, mutations are drawn from an assumed distribution that is centered on the phenotypic position of each individual (Fig. 1). By never descending into genotypic space, FGM is analogous to quantitative genetics models. Both types of models assume or require only that a component of phenotype is heritable. However, whereas quantitative genetic models represent populations by their mean and variance, FGM is an individual-based model in which each individual is evaluated according to its fitness.

FGM makes a set of theoretical predictions about how adaptation tends to occur, and many of these have been corroborated by experimental results. The greater frequency of small-sized beneficial mutations [12], the L-shaped distribution of mutations fixed throughout evolution [13], the existence of fitness equilibriums [14], and the absence of intrinsically beneficial or intrinsically deleterious mutations have all been observed during laboratory evolution, and all conform to the conditions set forth under FGM. The model thus appears to offer a reasonable framework for the study of microbial evolution.

The utility of FGM lies in the fact that it does not require any particular assumptions about the map between phenotype and genotype, and that the specific predictions about how fitness changes during evolution appear to be robust. In the present paper, we further refine previous predictions derived from FGM [9], [10] to take into account some fitness functions compatible with experimental data and use these results to estimate the phenotypic complexity of two viruses evolved in the laboratory.

## Model and Results

The link between drift load and phenotypic complexity under FGM was first investigated by Hartl and Taubes [9] (although Wagner and Gabriel [15] had worked previously on a similar question), and was later refined by Poon and Otto [10]. Recent theoretical techniques from statistical physics now allow an exact solution of FGM for several fitness functions. Sella and Hirsh [16] found that mean equilibrium fitness 〈f〉 can be written as
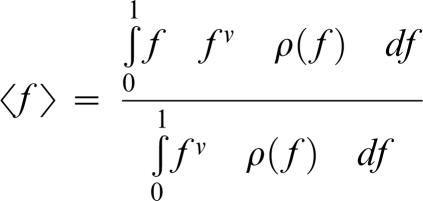



In which ν = 2⋅N_e_−1 in the diploid case and 2⋅N_e_−2 in the haploid, and ρ(f), the density function, *i.e.* the size of fitness f isocline, is dependent on the number of dimensions and of the fitness function used.

### Linear Fitness Decline

If fitness is assumed to be a linearly decreasing function of the phenotypic distance to the optimum, then we find that the average fitness is given by (Methods, Appendix A):
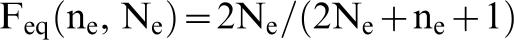
in the diploid case, in which F_eq_ is the equilibrium fitness (drift load) expressed as a fraction of the maximum attainable fitness of the organism, n_e_ is the *effective* number of dimensions of the phenotypic space (phenotypic complexity; see the below for a more detailed discussion of why we term this the effective number of dimensions), and N_e_ is the effective population size. This confirms the results obtained by Poon and Otto who approximated F_eq_(n_e_, N_e_) as 2N_e_/(2N_e_+n_e_) [10].

Although earlier studies on FGM model have used such linear fitness functions (for the sake of mathematical simplicity), recent experimental studies do not seem to support the use of such a function [17]–[19]. Linear fitness functions give rise to dramatic synergistic epistasis. For example, a mutation that increases the distance to the optimal phenotype by 0.1 units may decrease fitness by 10% in the optimal genotype (which by definition has a fitness of 1); an analogous mutation will decrease fitness by 50% in an organism with a fitness of 0.2; this mutation will become lethal in any genotype with a fitness less than 0.1. Recent experimental work suggests that epistasis between deleterious mutations is either antagonistic [17]–[19] or null [20]. We therefore decided to use a family of exponential fitness functions with a parameter that allows control over the level and shape of epistasis.

### Exponential-type Fitness Decline

In an effort to explore fitness functions more compatible with experimental data, we studied the following family of functions. f(d) = exp(−(d^Q^)), in which fitness is an exponentially decaying function of the distance to the optimum to the power of Q. Q is a parameter that modifies the concavity of the fitness decline. As organisms move away from the optimum the effect of the mutation tend to have bigger effect if Q>1 and smaller effect if Q<1. In such a case the fitness equilibriums are (Methods, Appendix A).
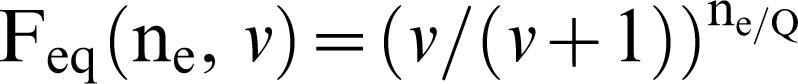



**Figure 2:**
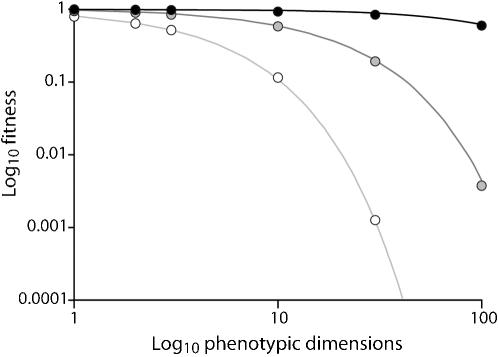
Predicted equilibrium fitness as a function of phenotypic complexity (n_e_). Results are shown for populations of size 100 (black), ten (grey), and three (white). An exponential fitness decline in which Q = 1 was used (yielding a fitness function of f(d) = exp(−d)). Circles indicate the average fitness reached in the simulation model; curves indicate the analytical results.

Thus in the haploid case on which we will focus later:
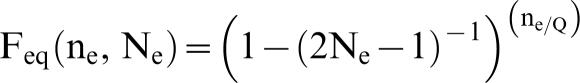



The validity of these results was confirmed by an individual based model of simulation analogous to one used previously [6] (Fig. 2).

### Robustness of Fitness Equilibrium to FGM Hypothesis

The implementation of FGM requires several assumptions in regards to the biology of the organism. The distributions of the mutations and the shape of the fitness function are required, and the geometry of the fitness isoclines needs to be symmetrical. However, we show below that the equilibrium drift load is fairly insensitive to these strict assumptions.

First, as equation (4) suggests, the fitness equilibrium is independent of the mutational properties. As long as mutation is assumed to be isotropic, only the convergence time to equilibrium, and not the equilibrium fitness value, is affected by the distribution of mutational effects (data not shown). Second, although the results depend on the shape of the fitness function (linear or exponential-type), they are independent of the slope: equilibrium values will be the same if f(d) = exp(−αd^Q^) (Methods, Appendix B). Third, although the canonical FGM assumes circular fitness isoclines, it can be shown that if fitness isoclines are elliptical instead of circular, then the density function is affected, but this cancels out in the calculation of fitness equilibrium. This holds for fitness isoclines and also for any fitness function of the form f(X) = exp(−∑α_i_x_i_^Q^), where X = (x_0_, x_1_, …, x_n_) is the coordinate of an individual in FGM, and α_i_ are positive parameters (Methods, Appendix B). Finally, if the mutation cloud is a Gaussian ellipsoid, it has been shown that an appropriate change of axes result in a space in which fitness isoclines are ellipsoid while the mutation cloud is circular [21]. Hence it seems that asymmetry in both the mutational distribution and the fitness isoclines do not affect the equilibrium fitness values, a robustness of the equilibrium fitness confirmed by some simulation data (data not shown).

The equilibrium drift load seems to be a robust property of FGM that is determined by the number of dimensions of phenotypic space, the population size and the fitness function (especially its curvature). An accurate estimate of phenotypic complexity can thus be obtained if it is possible to estimate equilibrium fitness values (drift load) for several population sizes, as well as the amount of curvature in the fitness function.

### Viral Evolution

**Figure 3:**
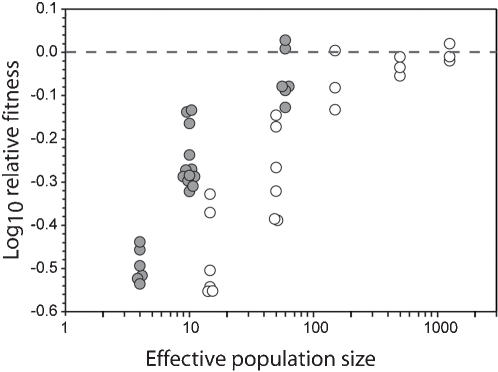
Equilibrium drift load as a function of population size for vesicular stomatitis virus and ΦX174. Each point indicates the mean fitness of a population. The VSV populations are shown in dark grey and the ΦX174 are shown in white. Some points have been displaced on the x-axis for clarity. The VSV populations were transferred at effective population sizes of four, ten, and 60; the ΦX174 populations were transferred at effective population sizes of 15, 50, 150, 500, and 1250. The dotted lines specify the maximum likelihood estimate of the f_ref_ value (the maximum attainable fitness); the dark dotted line indicates the value for VSV and the lighter dashed line indicates the value for ΦX174.

We used two sets of evolution experiments in which both fitness equilibrium values and fitness curvature have been investigated (Fig. 3). In the first set of experiments we evolved the bacteriophage ΦX174 on a bacterial lawn of its host, *Escherichia coli* C. We increased the mutation rate of the phage to hasten convergence to fitness equilibrium with the use of hydroxylamine; this resulted in a mean mutation rate of 0.1 per genome per generation through the 450 generations of evolution. Population size-dependent fitness equilibriums were observed, confirming that evolution occurred in a manner compatible with the assumptions of FGM. From these populations we obtained 21 measures of equilibrium fitness at five different population sizes. Every transfer corresponded roughly to five phage generations and effective population size was then approximated to five times the number of plaques transferred (the harmonic mean).

To estimate the curvature of the fitness function we performed a mutation accumulation analysis for high and low fitness clones and showed that the distribution of deleterious mutations was similar at both ends of the fitness range spanning a 300-fold difference. This suggests that there is very little curvature of the fitness function.

We used a second set of data from the literature, in which populations were evolved for 20 transfers at different effective sizes. Novella et al. [22] evolved four clones of vesicular stomatitis virus (VSV) using plaque-to-plaque transfers of sizes two, five, and 30. They also observed a population size-dependant fitness convergence. Although the time for convergence was relatively short (40 viral generations), several populations experienced no significant increases or decreases in fitness over all replicates at a given population size, suggesting that those population were at or near an equilibrium fitness value. We used the fitness estimates from 24 populations evolved at population sizes of two, five and 30. Every transfer corresponded to roughly two viral generations and effective population size was thus approximated as two times the number of viral plaques transferred.

Additionally, an impressive set of data using site directed mutagenesis in VSV suggests that the concavity of fitness function is slightly upward [19]. However, in further analyses we estimate Q as 1 for both the phage and the virus, as no clear departure from 1 has been observed in either case.

### Maximum Likelihood Estimate of Phenotypic Complexity

We wish to use the previous mathematical results to estimate phenotypic complexity from experimental data. However, there are two unknown parameters in the experimental system that affect the equilibrium drift load in a population: phenotypic complexity and the maximum attainable fitness that can be reached by the viruses in the laboratory environment (this parameter has been scaled to one in the previous derivations). Using methods from statistical physics, we can find the distribution of population fitness at equilibrium (shown above), and thus derive a likelihood model that gives the probability of the observed data for each couplet (n_e_/Q, f_ref_), in which n_e_ is the phenotypic complexity, Q a parameter of the curvature of the fitness surface, and f_ref_ the maximum attainable fitness. We also take into account the noise in our experimental assessment of fitness values; especially for high fitness populations, noise in the estimates of fitness can alter the estimation of f_ref_, as this parameter is by definition higher than all fitness measures. Thus rather than using the probability of the point estimate of fitness, we integrated the probability between plus (f_+_) and minus (f_−_) one standard deviation of the point estimate. In Appendix C we show that 
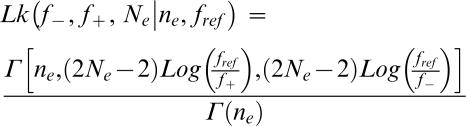



### Estimates of Phenotypic Complexity for VSV and ΦX174

We applied the maximum likelihood estimator to the experimental estimates of population fitness for ΦX174 and VSV, and using a likelihood ratio test we defined 95% confidence intervals (CI), which we list here in parentheses. For ΦX174 we found n_e_/Q = 45 (42−49), and f_ref_ = 1.245 (1.23−1.26), whereas for VSV we found n_e_/Q = 10 (8−12) and f_ref_ = 1.98 (1.94−2.05) (**Fig. 3**). In both of these cases, f_ref_ is calculated per generation relative to the ancestral virus for ΦX174 and relative to a reference strain for VSV. As no strong signature of curvature in the fitness surface has been found for either virus, we assume that Q is approximately one.

## Discussion

To understand how biological complexity changes during the course of evolution, a metric is needed. Previously, measures such as the number of genes, cell types or metabolic processes have been proposed, but they often lead to incongruent results. Organisms with more cell types do not necessarily have more genes. Here we suggest that a metric unifying biological systems has not been appropriately identified. To circumvent this problem, we have developed a metric of biological complexity termed phenotypic complexity (n_e_). We have quantified this metric in the viruses ΦX174 and VSV by utilizing a population genetic model that describes how phenotypic complexity affects the drift load that a population experiences.

Phenotypic complexity (n_e_) is a measure of the number of genetically uncorrelated phenotypes that are acted upon by natural selection. Because Darwinian evolution through natural selection is the fundamental element unifying all biological organisms, we propose that n_e_ is potentially a more relevant metric than those previously suggested.

### Robustness of the Model

Using recent theoretical results we have analytically quantified the dependency of the drift load (equilibrium fitness) on the effective population size and phenotypic complexity. The linear fitness function that has been employed previously to simplify the mathematical analyses is no longer necessary. Such a function makes strong assumptions about the form of the fitness landscape; specifically, mutational effects become very large as fitness is reduced. Thus at low fitness most mutations are either lethal or of very large effect, a scenario which is incompatible with what we have previously observed [14].

We have therefore studied a more general family of fitness functions of the form f(d) = exp(−(d^Q^)), and found that F_eq_(N_e_, n_e_) = (1−(2⋅N_e_−1)^−1^)^(ne/Q)^. It appears that this equation remains valid over a much wider range of conditions than those used in the canonical FGM, in which mutations are required to be isotropic and fitness isoclines are symmetric about the origin. An interesting feature of this formula is that it does not require a model in which mutations can affect all phenotypic traits simultaneously. In the initial formulation of FGM, all phenotypic axes intersect at the origin of each axis. This original FGM can be modified slightly such that some phenotypes are grouped into separate phenotypic modules, and within a module, all phenotypes again intersect at each other's origin. Any mutation that occurs within a module can affect only other phenotypes within that module, and none that lie outside of it (*i.e.* there is no pleiotropy between modules, an idea similar to previous conceptions of modularity [23]). Using the more general description of the equilibrium drift load that we have derived above, a phenotypic landscape a composed of a single module with n_e_ dimensions has the same drift load function as a landscape composed of m independent modules of size n_e,i_ with ∑_i_ n_e,i_ = n_e_ because we have 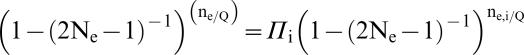



Hence the drift load formula that we have obtained seems to be robust to many of the assumptions underlying FGM.

### Other Attempts to Estimate Phenotypic Complexity

Recently, another theoretical study developed a framework to estimate phenotypic complexity [21]. The model developed used predictions on the distribution of mutational effects to estimate complexity. This analysis, which is completely independent from ours, found an interesting correlation between gene number and complexity in a variety of organisms ranging from a virus to *C. elegans*. However, for all the organisms for which enough data existed to perform the analysis, the number of phenotypic dimensions estimated was very small; 0.21 phenotypic dimensions for *E. coli*, 1.07 in VSV, 1–2 for *S. cerevisiae*, and 2–3 for *D. melanogaster* and *C. elegans*. We think that several effects may limit the predictive quality such a method. Firstly, mutation accumulation methods and the inherent noise in fitness estimates are very different across species and comparison across species are thus difficult. One illustration of this is the large variability in fitness estimates for mutations in a single species using two methods: in VSV earlier estimates (using Bateman-Mukai estimates) predicted a 0.002 mean fitness effect per mutation, while more recent and accurate estimates (which introduced each mutation individually) brought the estimate it to 14% (a 70-fold difference). Secondly, neutral mutations are not considered in the model developed by Martin and Lenormand. However, part of the mutation produced by transposable elements might affect genes useless in laboratory conditions and will therefore be taken into account in the calculation of mean fitness effects of mutations even if they do not affect any phenotype in the laboratory environment. Third, contrary to our model, if phenotypes are organized in different modules (as many data suggest), their model will provide different estimates; in other words, their model requires that all traits can be simultaneously affected by a single mutations. All these considerations suggest that a second independent model should be used to estimate phenotypic complexity.

One of the central FGM hypotheses that we have so far not addressed is the single-peaked nature of the landscape. Although FGM contains few assumptions about the nature of the genotypic landscape, the model explicitly requires a phenotypic landscape containing a single peak; without this, then the fitness function, f(d), cannot be described by a decreasing function. However, recent experimental evidence over large evolutionary time scales strongly suggests that while the genotypic landscape may contain multiple peaks, the phenotypic landscape is generally much less complex. Several experimental studies using microbes have shown that a considerable amount of phenotypic convergence occurs during evolution [24]–[27], even when organisms begin from different starting points in the landscape [28]. These phenomena would only be expected if the phenotypic landscape exhibited a single-peak. However, the evidence for ruggedness in the genetic landscape is also substantial, especially in experiments that have looked at bacterial evolution of resistance to antibiotics [29]. In this case, the fitness relationships between the wild type genotypes, resistant genotypes, compensated resistant genotypes, and sensitive genotypes bearing the compensatory mutation exhibit high levels of epistasis, characteristic of a rugged genetic landscape. In other studies in which the cost of resistance was associated with a phenotype [30], fitness restoration to wild-type level was sometimes observed and, more importantly for our concern, it was associated with a restoration of the phenotypic damage associated with the occurrence of the resistance mutation (transcription efficiency of rifampicin resistant mutants was restored back to the level observed in rifampicin sensitive strain). This also suggests the singularity of the phenotypic optimum, although different genetic combinations may underlie this optimum. However, as our model is focusing neither on the genetic nature of the adaptive landscape, nor on the rate of adaptation (ruggedness means that several mutations could be needed to restore the effect of one) it should not be too sensitive to the small level ruggedness of the genetic landscape described so far.

### The Concept of Phenotypic Complexity

As discussed previously, the quantity denoted by n_e_ is the number of genetically uncorrelated phenotypes that are influenced by the action of natural selection. The dimensions enumerated by n_e_ are thus genetically orthogonal to each other, and analogous to the axes needed to describe the variation among multiple phenotypes measured on a collection of individuals and mutants in a principal component analysis. However, the number of axes enumerated by n_e_ is filtered by natural selection, while in a PCA analysis the number of axes is limited only by the number of independent phenotypes that are measured. Because each phenotype is optimized at a value determined by each organism's ecological environment, there is a dependence of phenotypic complexity on the complexity of the ecological niche experienced by each organism; if natural selection does not act on a phenotype, then that phenotype does not contribute to the complexity metric. Finally, although the estimates of n_e_ arise from an idealized model of phenotypic evolution; as Orr suggested previously, estimates of phenotypic complexity using FGM can be viewed as “effective” estimates of phenotypic complexity [6]. This concept is analogous to the concept in population genetics of effective population size, N_e_, in which two populations with different numbers of individuals and different sex ratios might have the same effective population size and therefore respond similarly to the different population genetic forces. Thus two organisms, although they may differ in both the underlying genetic mechanisms and in the complexity of the environment in which they live, may have similar phenotypic complexities. The utility of the concept lies not in the implications it makes about specific phenotypes or genetic details, but in that it enables a general quantification of how an organism is affected by natural selection (the *complexity* with which natural selection acts), and furthermore, how this action affects the evolutionary dynamics of that organism [6].

### Viral Estimates of Phenotypic Complexity

Unsurprisingly, our estimates of phenotypic complexity are orders of magnitude smaller than either the number of nucleotides or even the number of amino acids encoded by the genomes of these organisms (5386 bp and 11,161bp in ΦX174 and VSV, respectively). This agrees with the concept of phenotypic complexity that we have defined. Although mutations that occur at one nucleotide or amino acid do not affect those at another (*i.e.* they are genetically separable phenotypes), it is clearly unlikely that each nucleotide or amino acid is independently acted up by natural selection. Secondly, each estimate of complexity is greater than the number of genes encoded by each virus (11 and 5 for ΦX174 and VSV, respectively). The presence of multiple functional domains within a single protein is consistent with an estimate of complexity that is greater than the number of encoded proteins.

Although we have only two estimates, we can briefly consider them from a comparative standpoint: although the genome size of ΦX174 is half of VSV, our estimate of phenotypic complexity quantifies ΦX174 as being approximately four-fold more complex. It is notable, then, that ΦX174 contains approximately twice the number of genes as VSV. Additionally, the lifestyle of ΦX174 is arguably much more elaborate than that of VSV. ΦX174 interacts with several host factors in order to perform transcription and replication; 13 host factors are required for replication alone [31]. This can be contrasted with VSV, in which transcription and replication are similar processes, both performed by the viral polymerase. Few host factors (and thus few interactions) are necessary at all during the entire life cycle of VSV [32].

These estimates suggest that, for very simple organisms such as viruses, phenotypic complexity correlates well with the number of genes in an organism, and more specifically, with the number of interactions characteristic of that organism [2]. This observation supports the idea that phenotypic complexity depends on the interactions between an organism and its environment. Genome size in itself seems to be a poor correlate of phenotypic complexity, a notable result in consideration of the very small genome sizes of these organisms. Whether such correlations or absence of correlation with gene number or genome length would hold for more complex organisms remains an open question. Nevertheless, by studying very simple organisms, we have shown that the use of drift load could be used to estimate complexity in a novel and consistent way. We now propose that such a method could be helpful to identify and quantify the strongest determinants of biological complexity of higher organisms.

Although the theory presented here appears to be quite robust, it is too early to conclude that it is an accurate reflection of the underlying biology. To be studied in an FGM framework, organisms need to present at least one phenotypic property to selection. Additionally, populations, even those of very small size, should evolve towards a fitness equilibrium that is explicitly dependent on population size. We found data in the literature consistent with this expectation for one organism, VSV. We now provide further support for population size-dependent fitness equilibria by evolving populations of the bacteriophage ΦX174. Together, these two data sets suggest that evolutionary analyses using an FGM framework are a valid approach. Moreover, the use of very simple organisms such as viruses is useful for gaining insight into metrics of complexity, as for such simple organisms, gene number is likely to be a very good correlate of organismal complexity, and this should be reflected by the metric. Although our observations are currently limited to two viral species, it is clear that from both a qualitative level (*i.e.* population size-dependent fitness equilibria), and a quantitative level (that the number of phenotypic dimensions are reasonable) that the predictions from FGM theory are borne out. An assessment of drift load and phenotypic complexity in a greater number of organisms is needed before further conclusions can be drawn.

### Conclusions

Here we have presented a top-down approach to quantifying biological complexity. This can be contrasted with previously proposed metrics of complexity, which have relied on physically measurable quantities of the organism (bottom-up approaches). Two important conceptual differences separate these two approaches. Most importantly, phenotypic complexity is dependent on both the organism and the environmental context. An organism is not complex because it has many measurable phenotypes; it is complex because it has many phenotypes on which natural selection acts. Secondly, phenotypic complexity does not rely on artificially constructed concepts such as genes [33]. As an example, if two genes are deemed to be of equal complexity because they are functionally equivalent, such a measure necessarily ignores the subtle ways in which each may be regulated, or spliced, or expressed within the cell. Quantifying such multiple layers of complexity is difficult if the metric relies on physically measurable quantities.

However, phenotypic complexity remains an inherently abstract metric. It cannot aid in identifying the specific characteristics contributing to the complexity of an organism. Instead, it addresses the complexity with which natural selection views an organism, and the complexity with which an organism is capable of generating novel phenotypic variation. For this reason, testing how phenotypic complexity compares to more traditional metrics of complexity (for example, the numbers of genes, protein interactions, or cellular pathways) may provide significant insight into biological systems. Finally, phenotypic complexity (and the resulting equilibrium drift load) affords a unique opportunity to contrast the action of natural selection between different organisms or different environments in a very general and unconstrained manner.

## Methods

### Viral Evolution

The details of experimental evolution of ΦX174 have been described previously [14]. Briefly, phage were mutagenized in 250 mM hydroxylamine, 1mM EDTA at 37°C for 140 minutes. Mutagenic treatment was stopped by 100 to 1000-fold dilution into fresh media, after which they were plated on LC agar plates containing a bacterial lawn and grown overnight at 32°C. From these plates, a number of plaques equal to the bottleneck size were randomly selected and diluted into culture tubes containing 3 ml of 1mM EDTA. These tubes were vortexed and centrifuged, after which 0.5 ml was removed to a fresh eppendorf tube. Chloroform was added, the tubes were vortexed and centrifuged, and 0.3 ml was removed. This stock was then used for further mutagenesis.

### Appendix A: Derivation of Fitness Equilibriums

Sella and Hirsh [16] showed that mean equilibrium fitness could be written
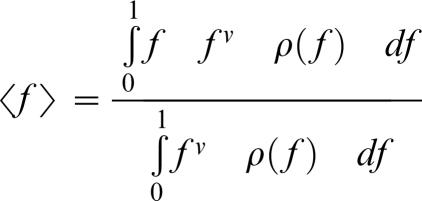
in which ν = 2N_e_−1 in the diploid case and 2N_e_−2 in the haploid, and ρ(f) is the density function of fitness value f.

In an n-dimensional space, the density ρ(*f*) d*f* is derived from the hyper-sphere surface of radius *x*, with f = f(*x*), f(*x*) being the fitness function describing the dependency of fitness on the distance to the optimum, *x*. The surface of the hyper-sphere of radius *x* is Ω(*n*)*x* ^(*n*−1)^, where Ω(*n*) is the unit radius hyper-sphere surface, Ω(*n*) = 2⋅π^(*n*/2)^/Γ(*n*/2). We have therefore ρ(*f*)d*f* = Ω(*n*) *x* ^(*n*−1)^ d*x*

If fitness is defined as f(*x*) = 1−*x*, then Ω(*n*) *x* ^(*n*−1)^ d*x* = −Ω(*n*) (1−*f*)*^n^* ^−1^ d*f* and
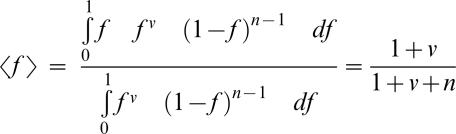



If fitness is defined as: f(*x*) = exp(−*x* ^Q^) we have 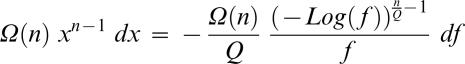
and 
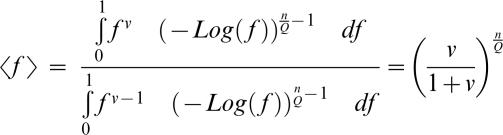



This expression is independent of mutational properties.

### Appendix B: robustness to assumptions

#### Slope of the fitness function

Note that if f(*x*) = exp(−α *x* ^Q^)
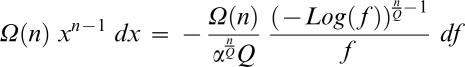
and 〈f〉is not affected.

#### Ellipsoidal fitness isoclines

Let us assume that f = exp(−*R* ^Q^) where *R* is defined by 
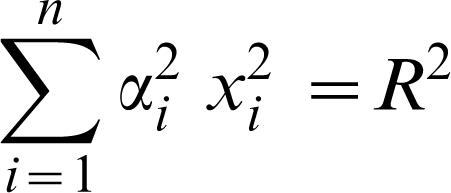
in which **x** = (*x*_1_,*x*_2_,…,*x*_n_) is the position in the n-dimensional space and α_i_ are positive numbers. We then have ellipsoidal fitness isoclines of semi-axes R/α_i_. As the volume of such an ellipsoid is 
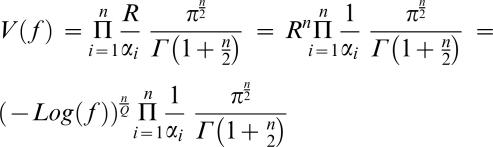
with 
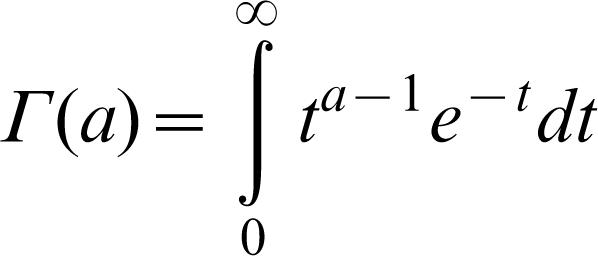
 being the Euler Gamma function and the density ρ(*f*) d*f* = dV(*f*) d*f* is similar to the one found in the previous case 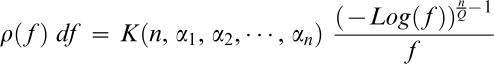
with
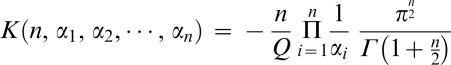



we therefore find the same value of 〈f〉, as the constant cancels out in the ratio of integrals. More generally, if fitness is defined as 
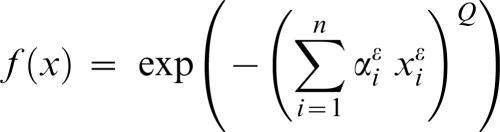



We can show through recursions that this defines volumes 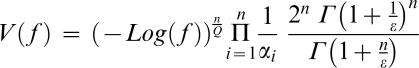
and that once again the equilibrium fitness remains unchanged.

### Appendix C: Maximum Likelihood Analysis

Sella and Hirsh showed that the probability of being at fitness *f* is 
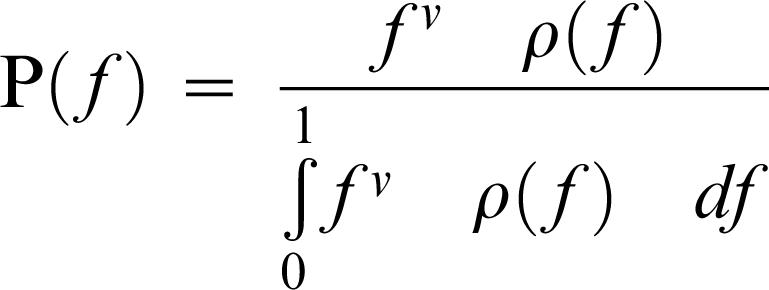



Using the previous derivations with f(*x*) = exp(−*x* ^Q^), we find the probability that *f* lies between a and b is
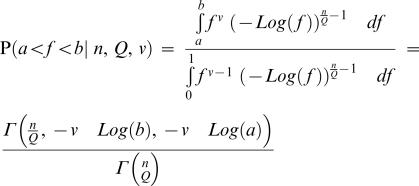
with 
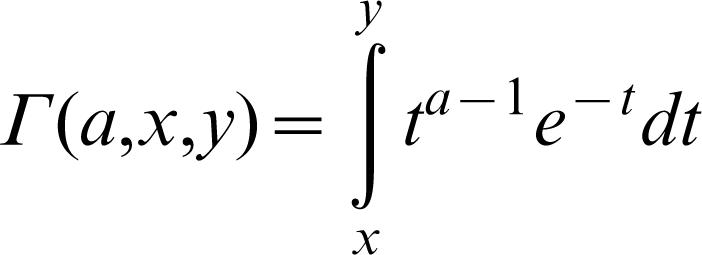
 being the generalized incomplete gamma function

Because we do not know maximum fitness f_ref_, we must estimate it and therefore fitness b and a be used relative to f_ref_.